# The Cumulative Variations of Respiratory Syncytial Virus Fusion Protein (F) in Ten Consecutive Years in China

**DOI:** 10.3390/idr16050081

**Published:** 2024-10-17

**Authors:** Fengjie Wang, Mingli Jiang, Zhenzhi Han, Yanpeng Xu, Yu Sun, Runan Zhu, Dongmei Chen, Qi Guo, Yutong Zhou, Yao Yao, Ling Cao, Dong Qu, Muya Li, Linqing Zhao

**Affiliations:** 1 Laboratory of Virology, Beijing Key Laboratory of Etiology of Viral Diseases in Children, Capital Institute of Pediatrics, Beijing 100020, China; wangfj1226_cdc@126.com (F.W.); jiangmingli00@126.com (M.J.);; 2 Department of Respiratory Medicine, Affiliated Children’s Hospital, Capital Institute of Pediatrics, Beijing 100020, China; 3 Department of Critical Care Medicine, Affiliated Children’s Hospital, Capital Institute of Pediatrics, Beijing 100020, China; 4 Friedman School of Nutrition Science and Policy, Tufts University, Boston, MA 02111, USA

**Keywords:** respiratory syncytial virus, fusion protein, antigenic sites, variations

## Abstract

Background: Variations in the fusion (F) protein of respiratory syncytial virus (RSV) with main antigenic sites I–V and Ø may affect the development of RSV vaccines and therapies. Methods: In the study, 30 respiratory specimens positive for RSV were randomly selected from children with acute lower respiratory infections (ALRI) in Beijing every year from 2012 to 2021 for *F* gene sequencing. Then, 300 *F* gene sequences and 508 uploaded to GenBank from China were subjected to phylogenetic analysis. Results: The results indicated the nucleotide identities were 95.4–100% among 446 sequences of RSV A, and 96.3–100% among 362 of RSV B. The most common variant loci were N80K (100.00%) and R213S (97.76%) for site Ø, and V384I/T (98.43%) for site I among sequences of RSV A, and M152I (100.00%), I185V (100.00%), and L172Q/H (94.48%) for site V, and R202Q (99.45%) for site Ø among sequences of RSV B. N276S appears in 95.29% sequences of RSV A, while S276N and N262 I/S appear in 1.38% and 0.55% sequences of RSV B, respectively. No variation was found in all sequences at the binding sites of 14N4 and motavizumab. Conclusions: There were cumulative variations of the RSV *F* gene, especially at some binding sites of antigenic sites.

## 1. Introduction

Human orthopneumovirus, also known as human respiratory syncytial virus (RSV), is the most common cause of acute lower respiratory infection (ALRI), with high morbidity and mortality among infants, young children, the elderly, and immunocompromised adults worldwide. In 2019, there were 3.6 million hospital admissions for RSV-ALRIs and 26,300 in-hospital RSV-ALRI deaths. Among children aged 0–6 months, there were 1.4 million RSV-ALRI hospital admissions, resulting in 13,300 deaths worldwide [1]. In China, the prevalence of RSV among children under 2 years of age with severe acute respiratory disease is 17–33% [2].

RSV is a single-stranded negative-sense RNA virus that belongs to the *orthopneumovirus* genus of the *pneumoviridae* family [3]. The RNA genome contains 10 genes encoding 11 proteins. The fusion glycoprotein (F) and attachment protein (G) are the two major RSV proteins that contain the main antigenic determinants associated with neutralizing antibodies. The sequence of the *F* gene is conserved with almost 90% identity between RSV A and B. Neutralizing antibodies induced by the F protein can simultaneously inhibit RSV A and B infection, so candidate vaccines for RSV development usually target the F protein [4]. There are two conformations of the F protein: pre-fusion (pre-F) and post-fusion (post-F). During the process of viral entry, F transitions from a metastable pre-F to a stable post-F conformation, which is necessary to initiate viral entry into host cells [5]. Six major neutralizing antigenic sites (I–V, and Ø) were defined to specific structural domains of pre-F [6]. Antigenic sites Ø and V are present only on pre-F and are targeted by highly effective RSV-neutralizing antibodies. Other sites (I, II, III, and IV) are found in both pre- and post-F conformations, whereas the effectiveness of neutralizing antibodies against these sites depends primarily on the accessibility and affinity of binding to pre-F [7].

Many vaccine strategies have been developed, including vector vaccines, subunit or particle vaccines, live attenuated vaccines, mRNA vaccines, and monoclonal antibodies (mAbs) [8,9]. In 2023, the USA Food and Drug Administration (FDA) approved three RSV vaccines, of which Arexvy and Abrysvo were suitable for adults over 60 years old, and Arexvy provided 82.6% protection against RSV-ALRI. Notably, Abrysvo also has a protective effect in pregnant women [10]. The mAb, nirsevimab (MEDI 8897) was approved by the European Union and United Kingdom in November 2022 and by the FDA on 17 July 2023 (Nirsevimab-alip, AstraZeneca Befotus, Cambridge, UK), which marked a major breakthrough in the prevention of RSV in the first RSV season for newborns and infants [11]. Passive prophylaxis with palivizumab, a humanized anti-F monoclonal antibody, is restricted to premature infants [12]. AM22, 5C4, 14N4, motavizumab, 101F, hRsv90, and AM14 are in clinical trials, and these mAbs are the most promising in infants or the elderly [13,14]. Both AM22 and 5C4 are specific for the antigenic site Ø with high binding affinity. Motavizumab and 14N4 interact with antigen site II, while 101F is a site IV-specific neutralizing mAb. Similarly, hRsv90 binds to antigen sites V and Ø with a low affinity, whereas AM14 binds to sites V and site IV [14,15,16,17]. Previous studies have shown that slight changes in the genomes of RSV and human rhinovirus can affect virulence and escape host immunity, which were beneficial for escaping antiviral agents and vaccines [18]. Therefore, it is necessary to continuously monitor the cumulative variations in RSV *F* in epidemic strains, especially the target sites of antibodies and drugs, to warn of escape from existing antibody drugs and provide theoretical support for new prevention strategies.

To understand the molecular evolutionary characteristics of RSV *F*, the *F* gene sequences prevalent in children in Beijing and those uploaded to GenBank from China during 2012 to 2021 were analyzed to reveal the accumulated variations in the *F* gene over ten consecutive years, especially on the main antigenic sites. This study will provide theoretical support for the development of RSV vaccines and antibodies.

## 2. Materials and Methods

### 2.1. Clinical Specimens

Clinical respiratory specimens were collected from children aged 0–14 years old diagnosed as ALRI who were hospitalized in the Affiliated Children’s Hospital, Capital Institute of Pediatrics (Beijing, China), from 1 January 2012 to 31 December 2021, for respiratory pathogen screening using a capillary electrophoresis-based multiplex PCR (CEMP) assay (Ningbo HEALTH Gene Technologies Ltd., Ningbo, China). In the CEMP assay, 15 primer pairs were used to detect 13 pathogens using human DNA and RNA as control. Signals from the 15 labeled PCR products were measured using fluorescence. Given by the kit instructions, the positions of pathogen amplicons are as follows: adenovirus 110.2/113.9 nt (represents different subtypes), influenza virus (Flu) A 105 nt (H3N2 244.9 nt, 2009H1N1 163.3 nt), human bocavirus 121.6 nt, parainfluenza virus 181.6 nt, chlamydia 190.5 nt, human metapneumovirus 202.8 nt, Flu B 212.7 nt, Mycoplasma pneumoniae 217 nt, human coronavirus 265.1 nt, human rhinovirus 129.6 nt and RSV 280.3 nt.

The type of specimens included nasopharyngeal swabs, nasopharyngeal aspirates, and bronchoalveolar lavage, the type of specimen collected for each child is one of three. Specimens were immediately sent to the laboratory. Upon arrival at the laboratory, each clinical specimen was processed immediately using viral transport medium (Yocon Biotechnology Co., Ltd., Beijing, China) in a Class II biosafety cabinet, and 500× *g* centrifugation for 10 min. Partial supernatant was used for viral nucleic acid extraction and CEMP assay, and the remaining was stored at −80 °C for later use.

In this retrospective study, 30 specimens per year were randomly selected from those positive for RSV under the following criteria: (1) collected from 1 January 2012 to 31 December 2021; (2) hospitalized children aged 0–14 years old diagnosed with ALRI; and (3) positive for RSV RNA in the CEMP assay. In order to eliminate the bias caused by clinical severity, we followed the principle of randomness in the selection of specimens.

This retrospective study was approved by the Ethics Committee of the Capital Institute of Pediatrics (approval number: SHERLLM2022027). The ethics committee decided that written informed consent was not required for RSV-positive specimens, which were the left-over for clinical test.

### 2.2. Nucleic Acid Extraction and Reverse Transcription

Total nucleic acid was extracted from supernatant of each selected specimen using the QIAamp MinElute Virus Spin Kit (Qiagen GmbH, Hamburg, Germany) according to the manufacturer’s instructions. The nucleic acids extracted from RSV-positive specimens were used as a template to synthesize cDNA using a conventional two-step reverse transcription reaction with random primers according to the manufacturer’s instructions. The M-MLV (200 U/μL) was used as reverse transcriptase (Invitrogen, Carlsbad, CA, USA). Ribonuclease Inhibitor (50 U/μL, Q069, TransGen Biotech, Beijing, China) and dNTP (10 mM, C068, TransGen Biotech) were added separately to the reaction mixture.

### 2.3. Polymerase Chain Reaction (PCR) for RSV Subtyping and F Gene Amplification

The cDNA obtained by reverse transcription was used as the PCR template for RSV subtyping. The primer sets used were as follows: forward (G1 5′-TGGGAC ACTCTTAATCAT-3′) and reverse primers (G2 5′-TGATTCCAAGCTGAGGAT-3′, G3 5′-GTTGTATGGTGTGTTTC-3′) [19]. PCR reactions were performed according to the kits 2×EasyTaq^®^ PCR SuperMix and manufacturer’s instructions (AS111-01; TransGen Biotech). RSV A amplification product was 250 bp and RSV B amplification product was 341 bp.

To amplify the full-length RSV *F* gene for phylogenetic analysis, nested PCR was carried out using outer primer pairs (F1 5′-TTGGCAATGAYA ATCTCAAC-3′, R1 5′-TYYTGTTTAACATRAAGTTTTG-3′) in the first round and inner primer pairs (F2 5′-TTGGCAATGAYAATCTCAAC-3′, R2 5′-YCTTCGYGACATATTGC-3′) in the second round under the reaction conditions: 94 °C for 5 min; 94 °C for 45 s, 50 °C for 45 s, 72 °C for 3.5 min, for 35 cycles; 72 °C for 7 min. The primers were designed based on the genome sequences of the RSV A strain ATCC VR26 (AY911262) and RSV B strain (KF826843) and synthesized by Invitrogen Trading Co., Ltd. (Shanghai, China). The PCR products were purified and sequenced by Sanger sequencing (Sino Geno Max Co., Ltd., Beijing, China).

All sequences were edited and assembled to obtain the full F-coding region nucleotide sequences, and then DNAstar (version 6.0; DNAstar Inc., Madison, WI, USA) was used to translate the nucleotide sequence into the amino acid sequence. All sequences were saved in the Fasta format.

### 2.4. RSV F Gene Sequences from China Downloaded from GenBank

All RSV *F*-coding region nucleotide and amino acid sequences in GenBank uploaded from China during 1 January 2012 to 31 December 2021 were downloaded using keywords “human orthopneumovirus”, “human respiratory syncytial virus”, “RSV”, and “China”. All sequences were saved in Fasta format.

### 2.5. Phylogenetic and Mutation Analysis

The F-coding region sequences of the prototype strains Long and CH18537, and predominant genotype ON1 (GenBank: PP845216.1) of RSV A and BA9 of RSV B (GenBank: PP832945.1) downloaded from GenBank were used as reference sequences for RSV A and B, respectively. All sequences were aligned using BioEdit v7.2 (IBIS Biosciences, Inc., Carlsbad, CA, USA) to reveal nucleotide and amino acid variations. The Python script was used to extract mutations and analyze whether they are synonymous or nonsynonymous mutation sites. The Bayesian inference method implemented in BEAST (version 1.10.4). The maximum clade credibility (MCC) trees were summarized using Tree Annotator (version 1.10.4). Genetic diversity analysis was also performed using MEGA X. The main antigen sites, I–V and Ø, and the target sites of the monoclonal antibodies, 14N4, motavizumab, 101F, and AM14, were marked according to references.

### 2.6. Selective Pressure Analysis

The online analysis tool (Datamonkey) was used to analyze the selection pressure on the RSV *F* gene. The selective pressure sites of the amino acid sequences (446 of subtype A and 362 of subtype B) were analyzed by single-likelihood ancestor counting, Fast Unconstrained Bayesian AppRoximation, fixed effects likelihood and Mixed Effects Model of Evolution methods.

## 3. Results

### 3.1. Datasets of RSV F-Coding Region Nucleotide and Amino Acid Sequences

In this retrospective study, 30 patients with ALRI and positive for RSV, ranging in age from 0 to 14 years, were enrolled every year during 1 January 2012 to 31 December 2021. Then 300 RSV-positive respiratory tract specimens were selected in ten consecutive years, and a total of 300 sequences (Beijing) of the F-coding region were obtained, including 145 belonging to subtype A and 155 belonging to subtype B.

Among 4793 RSV sequences uploaded from China to GenBank, 508 sequences containing the F-coding region were uploaded between 1 January 2012 and 31 December 2021, which included 301 belonging to subtype A and 207 to subtype B.

In total, 808 nucleotide and amino acid sequences of the F-coding region constituted the datasets for the phylogenetic analysis, including 446 for subtype A and 362 for subtype B.

### 3.2. Genetic Diversity Analysis of F Sequences

**Figure 1:**
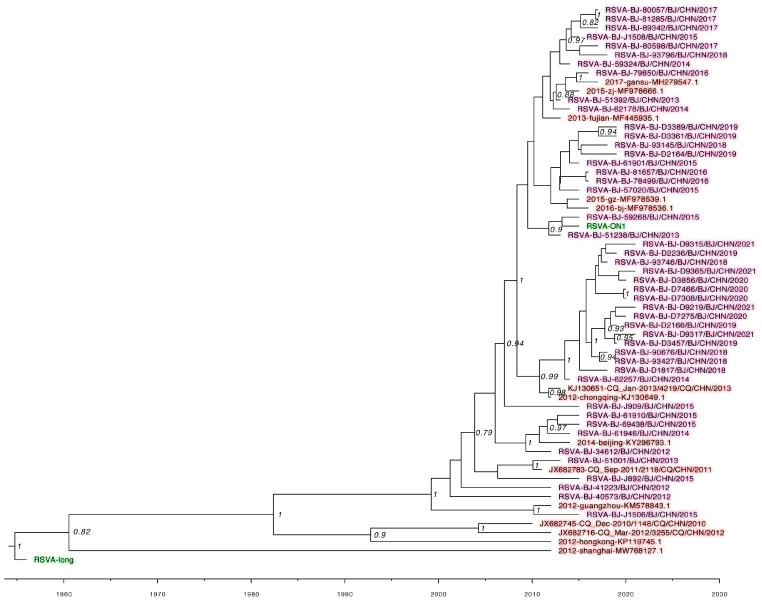
The Bayesian evolutionary analysis of the 60 representative RSV *F* gene sequences of RSV subtype A was constructed with BEAST software. The figure shows a detailed phylogenetic tree of the analyzed RSV A taxa. The maximum clade credibility (MCC) trees were summarized using Tree Annotator (only values over 70% are shown for clarity). The green shadows indicate the RSV A prototypes Long and ON1 (GenBank: PP845216.1) genotype strains. The pink shadows indicate the strains from this study during 2012–2021. The red shadows indicate the strains uploaded from China to GenBank.

**Figure 2:**
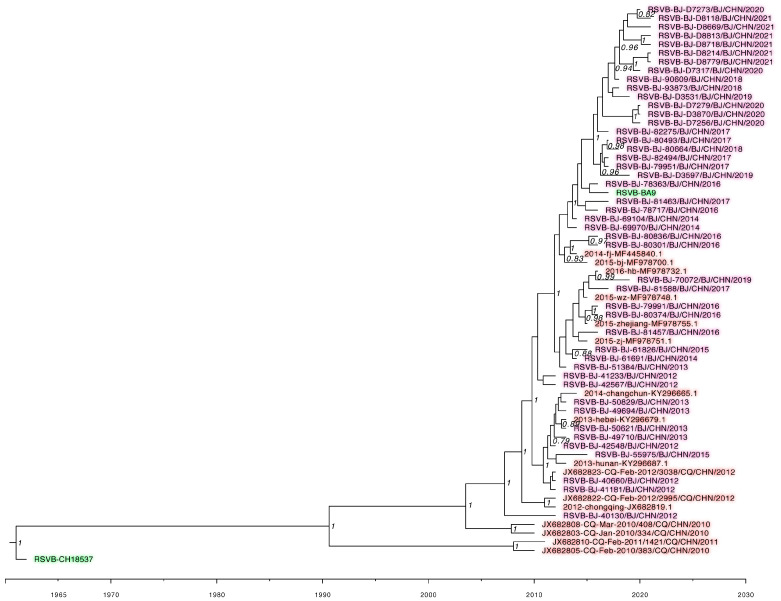
The Bayesian evolutionary analysis of the 60 representative RSV *F* gene sequences of RSV subtype B was constructed with BEAST software. The figure shows a detailed phylogenetic tree of the analyzed RSV B taxa. The maximum clade credibility (MCC) trees were summarized using Tree Annotator (only values over 70% are shown for clarity). The green shadows indicate the RSV B prototypes CH18537 and BA9 (GenBank: PP832945.1) genotype strains. The pink shadows indicate the strains from this study during 2012–2021. The red shadows indicate the strains uploaded from China to GenBank.

A total of 808 RSV *F* gene complete sequences, including 300 obtained (145 RSV A and 155 RSV B) in this study and 508 uploaded to GenBank from China during 1 January 2012 to 31 December 2021, were analyzed using sequences of strain Long and one belonging to genotype ON1 of RSV A, and CH18537 and one belonging to genotype BA9 of RSV B as reference sequences. RSV *F* gene phylogenetic trees are shown in Figure 1 and Figure 2. Compared with that of the prototype strains Long (RSV A) and CH18537 (RSV B), the mean distances were 0.012 for RSV A and 0.01 for sequences of RSV B. The nucleotide identity was 95.4–100% among 446 sequences of RSV A, and 96.3–100% among 362 sequences of RSV B.

### 3.3. Prediction of Selective Pressure Sites

The value of the nonsynonymous/synonymous substitution rate ratio (dN/dS) is an indicator of the intensity and mode of natural selection acting on protein-coding genes, and a dN/dS ratio > 1 is considered evidence of positive selection. The mean dN/dS ratios for RSV A and B were 0.138 and 0.165, respectively, indicating that the number of positively selected sites in the F sequence was much lower than that in the negatively selected sites. Using SLAC analysis, there was only one positive selection site (22aa) in subtype A, and no positive selection sites in subtype B. Using the FUBAR method, two positive selection sites (23aa and 105aa in subtype A, 21aa and 201aa in subtype B) were obtained, while two positive selection sites (3aa and 23aa) were predicted using the FEL method in sequences of subtype A. A total of three positive selection sites (16aa, 103aa, and 201aa) were predicted in the sequences of subtype B. Finally, the MEME method identified episodic and common positive selection sites, and there were five positive selection sites (3aa, 6aa, 23aa, 33aa, and 51aa) in the sequences of subtype A, and four positive selection sites (173aa, 201aa, 284aa, and400aa) in the sequences of subtype B (Table 1).

### 3.4. Analysis of Variations on Major Antigenic Sites of F

**Figure 3:**
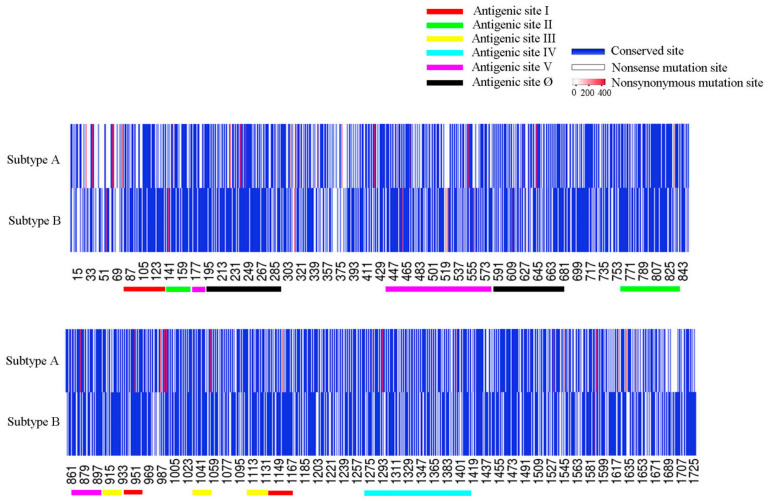
*F* gene nucleotide mutation in heat map using online software heatmapper (http://www.heatmapper.ca/), (accessed on 23 October 2023). In the heat map, the blue thin lines represent conserved sites, the white thin lines represent nonsense mutation sites, and the red thin lines represent nonsynonymous mutation sites. The number of sequences of meaningful mutation sites increases with the deepening of red, and the locations of different antigen sites are marked with thick colored lines.

Python script was applied to extract the mutations and analyze the mutation characteristics. It was found that compared with the sequences of Long (RSV A) and CH18537 (RSV B), the sequences of subtype A had 1073 nt absolutely conserved, 428 nt synonymous and 224 nt nonsynonymous mutation sites, whereas the sequences of subtype B had 1224 absolutely conserved, 320 synonymous and 181 nonsynonymous mutation sites (Figure 3).

There were 65 and 67 amino acid mutations in the six antigenic sites of the F proteins of RSV A and B, respectively. All amino acid mutations were caused by nucleotide substitutions, without nucleotide deletion or insertion mutations, and the mutation rate at most mutation sites was less than 5%. The variation rates greater than 0.3% are listed in Table 2.

The amino acid substitutions deduced from nucleotide sequences of RSV A were shown at the following major antigen sites: antigen site Ø (N80K, 100.00%), (R213S, 97.76%), I (V384I/T, 98.43%) and II (N276S, 95.29%), and the amino acid substitution of RSV B was mainly at antigen site Ø (R202Q, 99.45%; I206M, 30.39%; Q209K/R, 45.58%), I (F45L, 71.27%), and V (M152I, 100.00%; L172Q/H, 94.48%; S173L, 72.10%; I185V, 100.00%; K191R, 30.39%) (Table 2).

Among the sequences of subtype A shown in Table 3, there were 95.29% sequences with an amino acid residue substitution at the palivizumab binding site (N276S), and lower than 2.5% substitution rates shown in four residues (S169N, S173L, V178E, and K201N) at the hRsv90 binding site, while three residues (D200N, 2.47%; K201N, 2.02%; and K209R/Q, 2.02%) at the D25 binding site, two residues (D200N, 2.47%; and K209R/Q, 2.02%) at the MEDI 8897 binding site, two residues (K201N, 2.02%; and K209R/Q, 2.02%) at 5C4 binding site, and two residues (K209R/Q, 2.02%; and R213S, 97.76%) at AM22 binding site, were substituted. Among the sequences of subtype B, higher substitutions were observed at residues 202 (R202Q, 99.45%) and 209 (Q209K/R, 45.58%), which are common binding sites for D25, AM22, MEDI 8897, and 5C4, respectively. Compared with sequences of CH18537, 99.45%, sequences had substitutions at residue 202 (R→Q), 45.58% at 209 (Q→K/R), 72.10% at 173 (S→L) of hRsv90 binding site, 11.05% at residue 201 (N→S/K) of D25, 5C4 and hRsv90 binding site, and less than 1.5% of other binding sites (Table 3). The binding sites of antibodies 101F (I431T, 0.28%; K433Q, 0.28%; and S436F/C, 1.1%) and AM14 (N183Y, 0.28%; and N426T, 0.28%) were mutated only in RSV B.

## 4. Discussion

The *F* gene of RSV-endemic strains in China is relatively conserved and shows high homology, making it a potential candidate for the development of RSV *F*-targeting mAbs, vaccines, and drugs. Few molecular epidemiological studies have been conducted on RSV *F* infections in China. Zhou et al. [27] reported that F protein mutations mostly occurred in the NH-2 terminal, including antigenic site II, the target of palivizumab, containing one change, N276S, but the variation of important antigenic sites in F remains unclear. Nevertheless, many variations in *F* genes have been found in different clinical strains, especially between RSV subtypes. In this study, 300 RSV-positive respiratory tract samples were collected from children in Beijing from 2012 to 2021 for full-length F sequencing, combined with 508 RSV *F* gene sequences uploaded to GenBank from China, and comprehensively analyzed for variations in the *F* gene. The results indicated that RSV *F* was relatively conserved in China, and the sequence similarity of RSV A and B at the nucleotide level was greater than 95.4% and 96.3%, respectively. The sequence similarity of RSV A was slightly higher than that reported by Chen et al., whereas that of RSV B was similar to that reported by Chen et al. [28]. Kim et al. [29] reported that the nucleotide homology of the RSV *F* gene in Koreans during 1990–1999 was 93.5–98.7% in RSV A and 96.5–99.2% in RSV B. During 2006–2010, the average nucleotide homology of the *F* gene within subpopulations of RSV A and B was 97% and 98%, respectively [30]. However, the sequence similarity of RSV A and B reported by Xia et al. [31] was higher than that reported in a previous study, which may be due to temporal and geographical differences.

The RSV *F* gene has historically been relatively conserved, but continues to evolve [32]; however, there are limited studies regarding the antigenic variation and evolutionary patterns of RSV *F* genes. In this study, 65 and 67 amino acid mutations were identified in the *F* genes of RSV A and B, respectively. A study in the United States found that subtype B had a higher mutation rate at the F antigenic site than subtype A by analyzing samples from 2015 to 2017 [33], which resembles the results of the study showing that RSV B has more polymorphisms than RSV A. These results suggest that prevention or treatment based on the F of RSV A may not provide full protection against infections caused by RSV B, which is an important challenge for the development of mAbs or small-molecule drugs against RSV.

Previous studies have shown that the dN/dS values can be used to measure the strength and mode of natural selection acting on protein-coding genes [34]. In this study, the average dN/dS values of the *F* genes of RSV A and B were both <1, indicating that amino acid substitutions in the RSV *F* protein may be the result of random mutagenesis, and the difference between dN/dS ratios were partially related to differences in protein identity. Seven positive selection sites were identified for RSV A (aa3, 6, 22, 23, 33, 51, and 105) and seven sites for RSV B (aa16, 21, 103, 173, 201, 284, and 400) in 808 sequences of RSV *F*. Positive selection sites aa33 and aa51 were located in the F2 subunit, aa103 in P27 and aa173, 201, 284, and 400 were located in the F1 subunit. In addition, we found a 72.1% replacement of S173L and 11.05% replacement of N201S/K in the RSV B sequence. The potential impact of the substitution of these positive selection sites on the pathogenesis of RSV remains unclear.

Research and development of RSV vaccines is mainly aimed at the sequences of subtype A Long and subtype B CH18537. Therefore, these two strains were selected as reference strains for subtypes A and B, respectively, in the variation analysis of F. In this study, amino acid variants were observed in F1 (aa137–574), F2 (aa 26–109), and P27 (aa110–136) of RSV *F*, and frequently in F1, which may lead to changes in the secondary structure of the protein, altering the appearance of antigenic sites. In fact, variations in amino acid residues in all six epitopes were observed, particularly at the antigenic sites Ø and V, and these mutations have the potential activities to trigger the production of more efficient neutralizing antibodies [35]. Antigenic site V had three highly frequent amino acid variants, L172I/Q/H, S173L, and K191R, which were detected in strains of both subtypes A and B. Antigenic site I, an antigenic site that elicits a low-potency neutralizing antibody, is less likely to mutate [36]. However, a high frequency of V384I/T (98.43%) at antigenic site I of subtype A was observed in this study, with 97.76% V384I and only 0.67% V384T. These results are inconsistent with Prince [23], who reported the dominant wild-type amino acid change as V384T in South Africa. In subtype B, R202Q, M152I, L172Q/H, and I185V existing in antigenic sites Ø and V showed higher frequency (>90%). Some new low-frequency mutations, such as K68N, N208Y, and V178A/E have appeared which may provide a potential driving force for the evolution of RSV.

The effects of the widespread use of anti-RSV *F* mAbs on the emergence and spread of resistant variants are unclear. However, binding site variants may accumulate naturally under drug or immune selection pressure. In this study, the amino acid sequences of the binding sites for 14N4 and motavizumab remained conserved in both RSV A and B for ten consecutive years, similar to that for 101F and AM14 in RSV A. Therefore, these antibodies may provide broad anti-RSV effects in clinical application. Among the amino acid sequences of antigenic site II, the targets of palivizumab, were with N276S mutation in 95.29% of RSV A, 1.38% of RSV B, and with N262I/S in 0.55% of RSV B. Palivizumab blocks virus-cell and cell-to-cell fusion by binding to antigenic site II of F [11]. Mutations in residues 262, 272, and 275 of antigenic site II are important for palivizumab-resistant strains. However, neutralization tests have shown that N276S in RSV-A does not induce palivizumab resistance [37]. It has been reported that the mAb D25 fixes F in the conformation of pre-F by binding to the Ø antigenic site. In the binding region of the Ø antigenic site, three amino acid variations (D200N, K201N, and K209R/Q) were found in sequences of RSV A and six (K68N, N200D, N201S/K, R202Q, N208Y, and Q209K/R) in that of RSV B. McLellan et al. [20] also reported the sequence variations of the Ø antigenic site, the D25 binding region, but they did not find the variations of K68N, R202Q, and N208Y. Previous studies have demonstrated that AM22 and 5C4 potently neutralize RSV by preferentially binding to the pre-F conformation, similar to D25 [21,22]. The S211N polymorphism at antigenic site III has been detected in RSV B. The S173L mutations on antigen sites V and Ø interacting with hRsv90 [25] occurred in both RSV A and B, with higher frequency in RSV B than in RSV A, as well as N201S/K being more frequently found in RSV B, and S169N occurring only in RSV A. Whether these mutations will reduce the anti-RSV effects needs further study. The evolution and antigenicity of the virus are critical to the evaluation of existing vaccines and the development of new vaccines. As an important protein for RSV candidate antibodies, F is relatively conserved, but there are still many variations in the binding sites of candidate antibodies. These variants may have an impact on the clinical disease severity of RSV, such as the probability of co-infection and ICU admission, which may affect the immunological effectiveness of the vaccine. In addition, differences in variation between RSV A and B subtypes may contribute to differences in immunological efficacy of candidate vaccines across subtypes.

There were some limitations in this study. The number of specimens included in this study each year is limited and the types of specimens are not uniform, which may affect the results of the study. In addition, in the analysis of RSV subtype A, this study used strain Long as a reference strain rather than strain A2, which has also been considered for the design of RSV vaccines.

## 5. Conclusions

The accumulated variations in the RSV *F* gene over ten consecutive years, especially in the antigenic sites for mAbs, were analyzed in children with ALRIs. These results suggest that the *F* gene sequences of RSV A in China (including the nucleotide sequences of antigen sites) have been conserved for ten years, while those of RSV B were more prone to variation compared to the reference sequences. There were 95.29% of sequences with N276S substitutions at antigenic site II for palivizumab. New mutations, such as K68N, N208Y, and V178A/E, were also identified in this study. The genetic diversity of RSV *F* may affect the development of novel RSV vaccines and therapeutics. Therefore, it is necessary to continuously monitor the molecular evolution of RSV *F*.

## Figures and Tables

**Table 1:** Predicted selection pressure sites in RSV *F* sequences.

RSV Subgroup	Mean *dN*/*dS*	No. of Positive Selective Sites (aa Position)				No. of Negative Selective Sites			Significance *p*-Value
		SLAC	FUBAR	FEL	MEME	SLAC	FUBAR	FEL	
A	0.138	1 (22)	2 (23, 105)	2 (3, 23)	5 (3, 6, 23, 33, 51)	103	267	221	0.05
B	0.165	0	2 (21, 201)	3 (16, 103, 201)	4 (173, 201, 284, 400)	63	220	176	0.05

SLAC = single-likelihood ancestor counting, FUBAR = Fast, Unconstrained Bayesian AppRoximation, FEL = fixed effects likelihood, MEME = Mixed Effects Model of Evolution.

**Table 2:** Amino acid mutations at antigen sites of RSV *F* protein in China from 2012 to 2021.

Antigenic Site	RSV Subtype A (n = 446)		RSV Subtype B (n = 362)	
	V	N (F%)	V	N (F%)
Ø (62–96, 195–227)	N67T	9 (2.02%)	T67N/I	4 (1.10%)
	D73N/E	2 (0.45%) (2.47%)	T74A	3 (0.83%)
	A74T/V	18 (4.04%) (2.47%)	N197D	3 (0.83%)
	N80K	446 (100.00%)	N200D	3 (0.83%)
	D200N	11 (2.47%)	N201S/K	40 (11.05%)
	K201N	9 (2.02%)	R202Q	360 (99.45%)
	I206M/V	3 (0.67%)	I206M	110 (30.39%)
	K209R/Q	9 (2.02%)	Q209K/R	165 (45.58%)
	R213S	436 (97.76%)	S211N	2 (0.55%)
			R213S	3 (0.83%)
			K226M	2 (0.55%)
I (27–45, 312–318, 378–389)	E31A/K	2 (0.45%)	R42K	3 (0.83%)
	V40I	7 (1.57%)	F45L	258 (71.27%)
	K42R	3 (0.67%)	S380N	3 (0.83%)
	N380S	8 (1.79%)	T384I	3 (0.83%)
	V384I/T	439 (98.43%)	S389P	4 (1.10%)
	P389S	3 (0.67%)		
II (254–277)	N276S	425 (95.29%)	S259A	5 (1.38%)
			N262I/S	2 (0.55%)
			M274V	2 (0.55%)
			S276N	5 (1.38%)
III (46–54, 301–311, 345–352, 367–378)	Q302H	6 (1.35%)	I305L	3 (0.83%)
	L305I	9 (2.02%)		
IV (422–471)	Q462L	3 (0.67%)	S436F/C	4 (1.10%)
	S466N	13 (2.91%)	L456F/I	2 (0.55%)
			L462Q	3 (0.83%)
			N466S	4 (1.10%)
			L467F	2 (0.55%)
V (55–61, 146–194, 287–300)	E60K/A	2 (0.45%)	M152I	362 (100.00%)
	S150T	2 (0.45%)	N169S	3 (0.83%)
	S169N	11 (2.47%)	L172Q/H	342 (94.48%)
	L172Q/I	11 (2.47%)	S173L	261 (72.10%)
	S173L	9 (2.02%)	S180T	2 (0.55%)
	V185A/I	2 (0.45%)	I185V	362 (100.00%)
	K191R	2 (0.45%)	K191R	110 (30.39%)
	L297F	6 (1.35%)	E295G/D	2 (0.55%)

V = variation, N = number, F = frequency.

**Table 3:** The variation of binding sites of candidate antibodies targeting F protein.

Antibody Name	Antigenic Site	PDB	Binding Sites	References	RSV Subtype A (n = 446)		RSV Subtype B (n = 362)	
					V	N (F%)	V	N (F%)
D25	Ø	4JHA	63, 65, 66, 68, 200, 201, 202, 205, 208, 209	[20]	D200N	11 (2.47%)	K68N	1 (0.28%)
							N200D	3 (0.83%)
					K201N	9 (2.02%)	N201S/K	40 (11.05%)
							R202Q	360 (99.45%)
					K209R/Q	9 (2.02%)	N208Y	1 (0.28%)
							Q209K/R	165 (45.58%)
AM22	Ø	6DC4	202, 205, 209, 210, 211, 213, 215	[21]	K209R/Q	9 (2.02%)	R202Q	360 (99.45%)
							Q209K/R	165 (45.58%)
					R213S	436 (97.76%)	S211N	2 (0.55%)
							R213S	3 (0.83%)
MEDI8897	Ø	5UDD	65, 68, 200, 202, 208, 209	[11]	D200N	11 (2.47%)	K68N	1 (0.28%)
							N200D	3 (0.83%)
					K209R/Q	9 (2.02%)	R202Q	360 (99.45%)
							N208Y	1 (0.28%)
							Q209K/R	165 (45.58%)
5C4	Ø	5W24	63, 65, 68, 201, 202, 208, 209	[22]	K201N	9 (2.02%)	K68N	1 (0.28%)
							N201S/K	40 (11.05%)
							R202Q	360(99.45%)
					K209R/Q	9 (2.02%)	N208Y	1 (0.28%)
							Q209K/R	165 (45.58%)
14N4	II	5ITB	263, 269, 271	[14]	-	-	-	-
Palivizumab	II	-	262, 272, 275, 276	[23]	N276S	425 (95.29%)	N262I/S	2 (0.55%)
							S276N	5 (1.38%)
Motavizumab	II	3IXT	263, 269, 272	[13]	-	-	-	-
101F	IV	3O41	427, 429, 430, 431, 432, 433, 434, 435, 436	[24]	-	-	I431T	1 (0.28%)
							K433Q	1 (0.28%)
							S436F/C	4 (1.10%)
hRsv90	V, Ø	5TPN	169, 173, 174, 175, 178, 194, 201	[25]	S169N	11 (2.47%)	N169S	3 (0.83%)
					S173L	9 (2.02%)	S173L	261 (72.10%)
					V178E	1 (0.22%)	V178A	1 (0.28%)
					K201N	9 (2.02%)	D194Y	1 (0.28%)
							N201S/K	40 (11.05%)
AM14	IV, V	7MMN	160, 183, 426, 429	[26]	-	-	N183Y	1 (0.28%)
							N426T	1 (0.28%)

V = variation, N = number, F = frequency.

## Data Availability

The datasets used and/or analyzed in this study were obtained and available from the corresponding authors upon reasonable request.

## Abbreviations

RSV	respiratory syncytial virus
ALRI	acute lower respiratory tract infection
pre-F	pre-fusion
post-F	post-fusion
mAb	monoclonal antibody
FDA	the food and drug administration
ARTI	acute respiratory tract infection
CEMP	capillary electrophoresis-based multiplex PCR
dN/dS	nonsynonymous/synonymous substitution rate ratio
PDB	protein data bank

## References

[1] Li Y., Wang X., Blau D.M., Caballero M.T., Feikin D.R., Gill C.J., A Madhi S., Omer S.B., Simões E.A.F., Campbell H. (2022). Global, regional, and national disease burden estimates of acute lower respiratory infections due to respiratory syncytial virus in children younger than 5 years in 2019: A systematic analysis. Lancet.

[2] Yu J., Liu C., Xiao Y., Xiang Z., Zhou H., Chen L., Shen K., Xie Z., Ren L., Wang J. (2019). Respiratory Syncytial Virus Seasonality, Beijing, China, 2007–2015. Emerg. Infect. Dis..

[3] Vandini S., Biagi C., Lanari M. (2017). Respiratory Syncytial Virus: The Influence of Serotype and Genotype Variability on Clinical Course of Infection. Int. J. Mol. Sci..

[4] Sadoff J., De Paepe E., Haazen W., Omoruyi E., Bastian A.R., Comeaux C., Heijnen E., Strout C., Schuitemaker H., Callendret B. (2021). Safety and Immunogenicity of the Ad26.RSV.preF Investigational Vaccine Coadministered With an Influenza Vaccine in Older Adults. J. Infect. Dis..

[5] Saeland E., van der Fits L., Bolder R., Heemskerk-van der Meer M., Drijver J., van Polanen Y., Vaneman C., Tettero L., Serroyen J., Schuitemaker H. (2022). Immunogenicity and protective efficacy of adenoviral and subunit RSV vaccines based on stabilized prefusion F protein in pre-clinical models. Vaccine.

[6] Swanson K.A., Rainho-Tomko J.N., Williams Z.P., Lanza L., Peredelchuk M., Kishko M., Pavot V., Alamares-Sapuay J., Adhikarla H., Gupta S. (2020). A respiratory syncytial virus (RSV) F protein nanoparticle vaccine focuses antibody responses to a conserved neutralization domain. Sci. Immunol..

[7] Phung E., Chang L.A., Morabito K.M., Kanekiyo M., Chen M., Nair D., Kumar A., Chen G.L., Ledgerwood J.E., Graham B.S. (2019). Epitope-Specific Serological Assays for RSV: Conformation Matters. Vaccines.

[8] Gatt D., Martin I., AlFouzan R., Moraes T.J. (2023). Prevention and Treatment Strategies for Respiratory Syncytial Virus (RSV). Pathogens.

[9] Rezende W., Neal H.E., Dutch R.E., Piedra P.A. (2023). The RSV F p27 peptide: Current knowledge, important questions. Front. Microbiol..

[10] Mullard A. (2023). FDA approves long-acting RSV antibody. Nat. Rev. Drug Discov..

[11] (2023). Nirsevimab: First Approval. Drugs.

[12] Garegnani L., Styrmisdóttir L., Roson Rodriguez P., Escobar Liquitay C.M., Esteban I., Franco J.V. (2021). Palivizumab for preventing severe respiratory syncytial virus (RSV) infection in children. Cochrane Database Syst. Rev..

[13] Sun M., Lai H., Na F., Li S., Qiu X., Tian J., Zhang Z., Ge L. (2023). Monoclonal Antibody for the Prevention of Respiratory Syncytial Virus in Infants and Children: A Systematic Review and Network Meta-analysis. JAMA Netw. Open.

[14] Mousa J.J., Sauer M.F., Sevy A.M., Finn J.A., Bates J.T., Alvarado G., King H.G., Loerinc L.B., Fong R.H., Doranz B.J. (2016). Structural basis for nonneutralizing antibody competition at antigenic site II of the respiratory syncytial virus fusion protein. Proc. Natl. Acad. Sci. USA.

[15] ZhZhu Q., McLellan J.S., Kallewaard N.L., Ulbrandt N.D., Palaszynski S., Zhang J., Moldt B., Khan A., Svabek C., McAuliffe J.M. (2017). A highly potent extended half-life antibody as a potential RSV vaccine surrogate for all infants. Sci. Transl. Med..

[16] McLellan J.S., Chen M., Kim A., Yang Y., Graham B.S., Kwong P.D. (2010). Structural basis of respiratory syncytial virus neutralization by motavizumab. Nat. Struct. Mol. Biol..

[17] Sesterhenn F., Yang C., Bonet J., Cramer J.T., Wen X., Wang Y., Chiang C.-I., Abriata L.A., Kucharska I., Castoro G. (2020). De novo protein design enables the precise induction of RSV-neutralizing antibodies. Science.

[18] Moore M.L., Stokes K.L., Hartert T.V. (2013). The impact of viral genotype on pathogenesis and disease severity: Respiratory syncytial virus and human rhinoviruses. Curr. Opin. Immunol..

[19] Deng J., Qian Y., Zhu R.N., Wang F., Zhao L.Q. (2006). Surveillance for respiratory syncytial virus subtypes A and B in children with acute respiratory infections in Beijing during 2000 to 2006 seasons. Zhonghua Er Ke Za Zhi.

[20] McLellan J.S., Chen M., Leung S., Graepel K.W., Du X., Yang Y., Zhou T., Baxa U., Yasuda E., Beaumont T. (2013). Structure of RSV fusion glycoprotein trimer bound to a prefusion-specific neutralizing antibody. Science.

[21] Jones H.G., Battles M.B., Lin C.C., Bianchi S., Corti D., McLellan J.S. (2019). Alternative conformations of a major antigenic site on RSV F. PLoS Pathog..

[22] Tian D., Battles M.B., Moin S.M., Chen M., Modjarrad K., Kumar A., Kanekiyo M., Graepel K.W., Taher N.M., Hotard A.L. (2017). Structural basis of respiratory syncytial virus subtype-dependent neutralization by an antibody targeting the fusion glycoprotein. Nat. Commun..

[23] Mabilo P., Mthiyane H., Simane A., Subramoney K., Treurnicht F.K. (2022). Characterisation of RSV Fusion Proteins from South African Patients with RSV Disease, 2019 to 2020. Viruses.

[24] McLellan J.S., Chen M., Chang J.-S., Yang Y., Kim A., Graham B.S., Kwong P.D. (2010). Structure of a major antigenic site on the respiratory syncytial virus fusion glycoprotein in complex with neutralizing antibody 101F. J. Virol..

[25] Mousa J.J., Kose N., Matta P., Gilchuk P., Crowe J.E. (2017). A novel pre-fusion conformation-specific neutralizing epitope on the respiratory syncytial virus fusion protein. Nat. Microbiol..

[26] Gilman M.S.A., Moin S.M., Mas V., Chen M., Patel N.K., Kramer K., Zhu Q., Kabeche S.C., Kumar A., Palomo C. (2015). Characterization of a Prefusion-Specific Antibody That Recognizes a Quaternary, Cleavage-Dependent Epitope on the RSV Fusion Glycoprotein. PLoS Pathog..

[27] Zhou X., Jiang M., Wang F., Qian Y., Song Q., Sun Y., Zhu R., Wang F., Qu D., Cao L. (2023). Immune escaping of the novel genotypes of human respiratory syncytial virus based on gene sequence variation. Front. Immunol..

[28] Chen X., Xu B., Guo J., Li C., An S., Zhou Y., Chen A., Deng L., Fu Z., Zhu Y. (2018). Genetic variations in the fusion protein of respiratory syncytial virus isolated from children hospitalized with community-acquired pneumonia in China. Sci. Rep..

[29] Kim Y.K., Choi E.H., Lee H.J. (2007). Genetic variability of the fusion protein and circulation patterns of genotypes of the respiratory syncytial virus. J. Med. Virol..

[30] Papenburg J., Carbonneau J., Hamelin M., Isabel S., Bouhy X., Ohoumanne N., Déry P., Paes B.A., Corbeil J., Bergeron M.G. (2012). Molecular evolution of respiratory syncytial virus fusion gene, Canada, 2006-2010. Emerg. Infect Dis..

[31] Xia Q., Zhou L., Peng C., Hao R., Ni K., Zang N., Ren L., Deng Y., Xie X., He L. (2014). Detection of respiratory syncytial virus fusion protein variants between 2009 and 2012 in China. Arch Virol..

[32] Adhikari B., Hassan F., Harrison C.J., Bard J.D., Dunn J., Kehl S., Selvarangan R. (2022). A multi-center study to determine genetic variations in the fusion gene of respiratory syncytial virus (RSV) from children <2 years of age in the U.S. J. Clin. Virol..

[33] Lu B., Liu H., Tabor D.E., Tovchigrechko A., Qi Y., Ruzin A., Esser M.T., Jin H. (2019). Emergence of new antigenic epitopes in the glycoproteins of human respiratory syncytial virus collected from a US surveillance study, 2015–2017. Sci. Rep..

[34] Xu S., Zhang Y., Zhu Z., Liu C., Mao N., Ji Y., Wang H., Jiang X., Li C., Tang W. (2013). Genetic characterization of the hemagglutinin genes of wild-type measles virus circulating in china, 1993-2009. PLoS ONE.

[35] Krarup A., Truan D., Furmanova-Hollenstein P., Bogaert L., Bouchier P., Bisschop I.J.M., Widjojoatmodjo M.N., Zahn R., Schuitemaker H., McLellan J.S. (2015). A highly stable prefusion RSV F vaccine derived from structural analysis of the fusion mechanism. Nat. Commun..

[36] Huang J., Diaz D., Mousa J.J. (2019). Antibody Epitopes of Pneumovirus Fusion Proteins. Front. Immunol..

[37] Zhu Q., Patel N.K., McAuliffe J.M., Zhu W., Wachter L., McCarthy M.P., Suzich J.A. (2012). Natural polymorphisms and resistance-associated mutations in the fusion protein of respiratory syncytial virus (RSV): Effects on RSV susceptibility to palivizumab. J. Infect. Dis..

